# The Significance of Lignocellulosic Raw Materials on the Pore Structure of Activated Carbons Prepared by Steam Activation

**DOI:** 10.3390/molecules29133197

**Published:** 2024-07-05

**Authors:** Li Zhang, Songlin Zuo

**Affiliations:** 1 International Innovation Center for Forest Chemicals and Materials, College of Chemical Engineering, Nanjing Forestry University, Nanjing 210037, China; zlvol@njfu.edu.cn; 2 Jiangsu Co-Innovation Center of Efficient Processing and Utilization of Forest Resources, Nanjing Forestry University, Nanjing 210037, China

**Keywords:** activated carbon, steam activation, lignocellulosic materials, vitamin B12 adsorption

## Abstract

Five different lignocellulosic raw materials (coconut shells, Moso bamboo, sawtooth oak, Chinese fir, and Masson pine) were used to prepare activated carbons by steam activation at 850 °C to evaluate the effects of their structures on physical activation. The chemical compositions, botanic forms, and pore structures of the lignocellulose-based charcoal samples were systematically characterized by proximate and ultimate analyses, scanning electron microscopy, and mercury injection porosimetry. It was found that the rate of the activation reaction between charcoal and steam is determined by the porosity of the precursor. Pore structure results show that the steam activation of coconut shell and bamboo charcoals primarily produced micropores, thus yielding microporous activated carbon materials with just a few mesopores, even following a high burn-off of >66%. The steam activation of sawtooth oak charcoals produced mainly micropores at a low burn-off of <50% and both micropores and mesopores at a high burn-off of >50%. The steam activation of Chinese fir and Masson pine charcoals produced mainly mesopores at a burn-off of 0–80%. These mesopores were remarkably broadened to >20 nm on extending the activation time, resulting in a high vitamin B12 (VB12) adsorption capacity of ~530 mg/g. In conclusion, the raw lignocellulosic materials used as precursors have a decisive effect on the development of pore structures in activated carbon materials obtained through physical activation.

## 1. Introduction

Activated carbon materials are widely used in multiple fields, such as gas purification, wastewater treatment, electrocatalysis, batteries, and the pharmaceutical and food industries [1,2], due to their high specific surface areas, well-developed pore structures, good chemical stability, and abundant surface chemical groups. Essentially, the application performance of an activated carbon material depends mainly on its pore structure and surface chemical properties [3]. Activated carbon materials are usually fabricated in the industry by two approaches, namely physical activation and chemical activation, from a variety of raw materials, which include carbonaceous renewables, coal, and synthetic polymers [4]. The physical activation approach involves the creation of pores in the carbon matrix through gasification reactions of carbon atoms at high temperatures with steam, CO_2_, or air. The chemical activation approach is to create pores by mixing carbonaceous precursors with chemical activation agents such as ZnCl_2_ [5], H_3_PO_4_ [6,7], and KOH [8,9], followed by the steps of heat treatment and chemical recovery.

In physical activation in the industry, steam is commonly used as the activating agent, due to its high activation efficiency and given that it is an easily controllable activation process. Indeed, steam activation has been widely applied for nearly 100 years in the industrial manufacturing of activated carbon materials. Therefore, this traditional physical activation process has been extensively studied over the past few decades with regard to its activation mechanisms and the factors that influence the yield, pore development, and physicochemical properties of activated carbon products. These factors include the species of the raw materials [10], the activation agent [11], the activation temperature and time [12], the activation furnace [13,14], and the carbonization temperature of the carbonaceous precursor [15,16,17]. It is well recognized that physical activation produces a predominantly microporous structure, although physical activation with high burn-off of the char can produce some mesopores with a size of <5 nm. Previous investigations have consistently shown that physical activation using steam or carbon dioxide generates micropores or mesopores with a small size [18]. Besides coal, a variety of renewable lignocellulosic materials, including softwood [19,20], hardwood [21], nutshells [22], and bamboo [14,23], have been exploited as starting materials to produce activated carbon materials by physical activation. In previous investigations, although many lignocellulosic materials have been examined for the preparation of activated carbon by physical activation, their effects on the resultant pore structure have seldom been addressed. For this study, we selected five species of lignocellulosic materials to elucidate, for the first time, the effects of the botanical and physical properties of biomass-based chars on pore development. These are common starting materials for steam activation and are representative of nutshells, softwoods, hardwood, and bamboo. Accordingly, they have different botanic structures with regard to cell shapes, sizes, and arrangements. This investigation demonstrates that the original botanic textures of renewable materials have a decisive effect on the micropore or mesopore development of activated carbon materials obtained by physical activation. Activated carbon materials with a highly developed mesopore structure with a predominant size of 10–25 nm were prepared from suitable lignocellulose raw materials by steam activation. This investigation provides new insights into the regulation of pore structures in such traditional physical activations.

## 2. Results and Discussion

### 2.1. Properties of the Raw Materials

#### 2.1.1. Proximate and Ultimate Analysis of Charcoals

The proximate and ultimate analysis results of the raw charcoals are listed in Table 1. Evidently, the ash, volatile matter, and fixed carbon contents of these charcoal samples varied greatly with the species. Previous investigations have demonstrated that these parameters [24,25,26] have a significant impact on the development of pores during physical activation. The coconut (*Cocos nucifera*) shell charcoals (CSs), Chinese fir (*Cunninghamia lanceolata*) charcoals (FCs), and Masson pine (*Pinus massoniana*) charcoals (MCs) had much lower ash contents than the sawtooth oak (*Quercus acutissima*) charcoals (OCs) and Moso bamboo (*Phyllostachys edulis*) charcoals (BCs). Nevertheless, their C, H, O, and N contents were approximately the same.

#### 2.1.2. Environmental Scanning Electron Microscope (ESEM) Images of Charcoals

**Figure 1:**
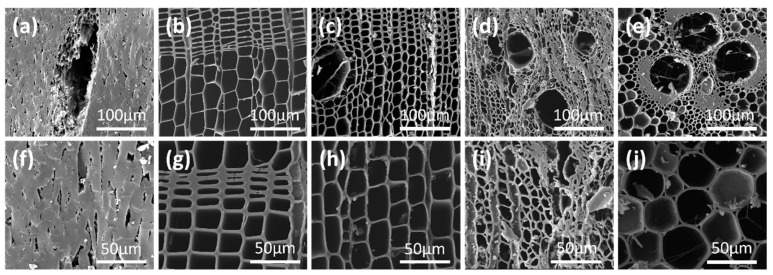
ESEM images of coconut shell (**a**,**f**), Chinese fir (**b**,**g**), Masson pine (**c**,**h**), sawtooth oak (**d**,**i**), and bamboo (**e**,**j**) charcoals.

Figure 1 depicts ESEM images of CSs, FCs, MCs, OCs, and BCs. All samples displayed micrometer-sized pores. The fracture surface of CS shows that the char contained vascular bundle pores and slit-like pores [27,28]. These pores were of various sizes and irregular shapes, and some may have been interconnected, serving as channels for the activation agent to enter the interior of the charcoal. Cross-sections of softwoods show resin canals mainly in latewood, large cavities and thin walls in earlywood, and small cavities and thick walls in latewood (Figure 1b,c,g,h). Longitudinal tracheid makes up the main cellular structure of softwoods, occupying about 90% of the whole wood volume [29]. Therefore, the activation reaction of coniferous charcoal occurs mainly on the tracheid. The size of the tracheid was 10–30 μm for both MCs and FCs, and their average wall thicknesses are ~1.5 μm and ~1.8 μm. Cross-sections of OCs show vessel pores with diameters of around 50 μm and tip cross-sections of wood fibers with pore diameters in the range of 5–25 μm (Figure 1d,i). Electron micrographs of BCs reveal xylem vessels with diameters in the range of 80–100 μm, parenchyma cells with diameters ranging from 15 to 25 μm, and an average wall thickness of ~2 μm, and dense bamboo fibers with small lumens and thick walls [30] (Figure 1e,j). The bamboo consisted mainly of longitudinal cell tissues, lacking transverse tissues. 

#### 2.1.3. Mercury Injection Porosimetry (MIP) Results

**Figure 2:**
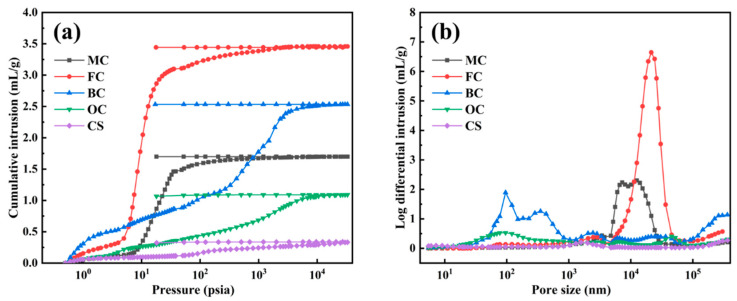
Mercury intrusion/extrusion (**a**) and pore-size distribution (**b**) curves of charcoal samples.

MIP measurement is the main method for characterizing pore structures in the size range from tens of nanometers to micrometers [31,32]. The results of MIP measurements are shown in Table 2, and mercury intrusion/extrusion and pore-size distribution plots are shown in Figure 2a and Figure 2b, respectively. Table 2 shows that the porosities of the charcoal samples decreased in the order of FC > BC > MC > OC > CS, in agreement with the order of total intrusion volumes [33]. Previous research has shown that the porosity of charcoal plays an important role in its activation process [34,35]. Here, FCs showed relatively abundant macropores of a size of about 20 μm, and MCs showed mainly macropores of 3–30 μm, which were probably derived from tracheid lumens, pits, or cracks [32]. BCs showed mainly pores in the size range of 0.025–1 μm, which were probably derived from pits as well as pores in the cell walls. Pore structures with a size distribution of 60–360 μm were also evident, which were probably derived from xylem vessels. The pore-size distribution of OCs showed peaks at around 0.09 μm and 45 μm, which is most likely attributable to small cracks in the cell walls and vessel pores, respectively. Compared with the other four charcoal samples, no obvious pore size peak was discernible for CSs, and the distribution was mainly concentrated at around 1.5 μm and 360 μm, respectively.

### 2.2. Progression of Charcoal Burn-Off with Activation Time

**Figure 3:**
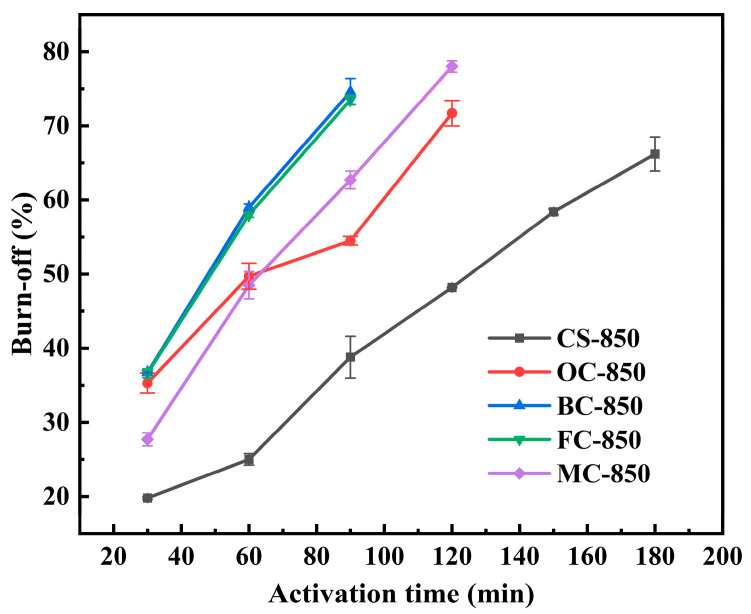
Variation of burn-off with the activation time for the five charcoal samples.

Figure 3 shows the progression of charcoal burn-off with an increasing activation time in the process of steam activation at 850 °C. Clearly, CSs exhibited the least burn-off, OCs and MCs showed moderate burn-off, and BCs and FCs showed the highest burn-off. This sequence is highly consistent with their porosities; the higher the porosity, the higher the burn-off [36]. Specifically, the CSs had a very low porosity of 29.10%, and the burn-off was only 66.2% after an activation time of 180 min. The porosities of FCs and BCs were 75.77% and 70.21%, and their burn-off extents exceeded 73% after an activation time of just 90 min. Table 1 shows that CSs and OCs had much higher volatile matter contents of 21.75% and 27.35%, respectively, as compared to less than 7.2% for the other three samples. Therefore, it is clear that the porosity of the starting charcoal is a decisive factor in determining the degree of burn-off in the process of steam activation. It is understandable that higher porosity provides more surface area for the activation reaction of charcoal with steam. We noted that OCs showed a much higher burn-off than MCs after activation for 30 min, possibly due to their much higher volatile matter content. After 60 min of activation, the higher burn-off of MCs than that of OCs can be ascribed to their higher porosity.

### 2.3. Development of Pore Structure at Burn-Off of Less Than 50%

In the process of physical activation with burn-off of the charcoal, pore development commonly involves four stages. The initial stage is the opening of closed pores in the raw charcoals by pyrolytic tar, which is associated with a low degree of ablation, typically less than 10%. The second stage is the creation of micropores, which is associated with a 10–50% burn-off of the charcoal. The third stage mainly involves extension of the micropores leading to the development of mesopores, typically in the burn-off range of 50–80%. In the final stage, the existing pores merge and collapse due to the considerable burn-off of the charcoal of more than 80%, and as a result, the surface area of the obtained activated carbon begins to decrease [23,37]. Accordingly, it can be recognized that physical activation involves micropore development at a low burn-off and mesopore development at a high burn-off [38,39]. A higher activation temperature was not used in this study, because charcoal reacts vigorously with steam, resulting in serious burn-off of charcoal.

**Figure 4:**
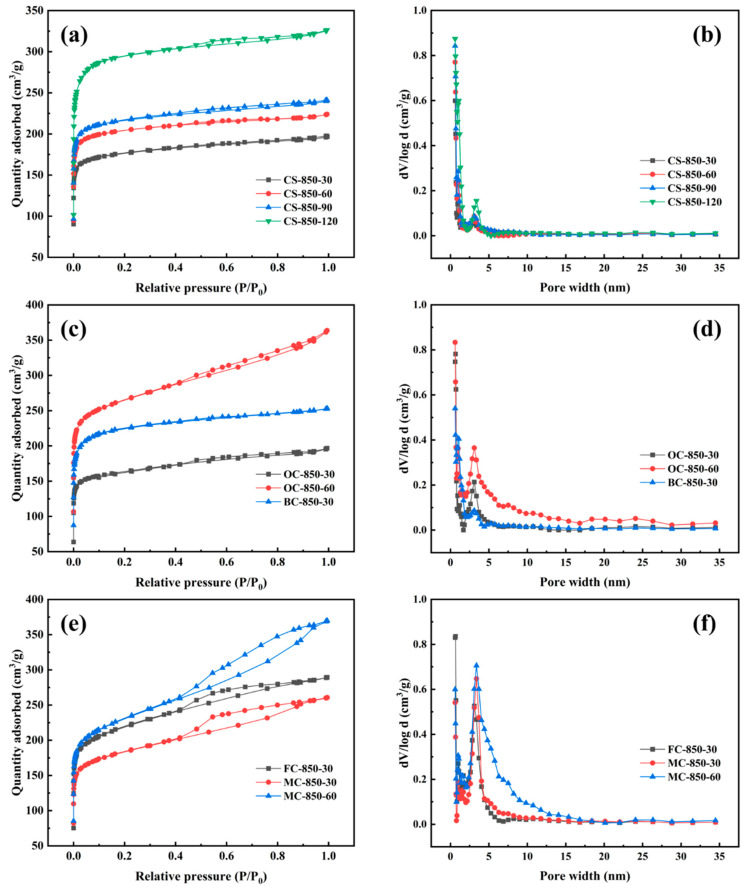
Nitrogen adsorption/desorption isotherms (**a**,**c**,**e**) and pore-size distributions (**b**,**d**,**f**) of activated carbons obtained at a low burn-off of <50%.

Figure 4a,c,e display nitrogen adsorption/desorption isotherms of CS-based activated carbons, BC- and OC-based activated carbons, and FC- and MC-based activated carbons obtained by steam activation at a lower burn-off of <50%. Figure 4a shows that the CS-based activated carbon samples prepared at a low burn-off were type I adsorbents without hysteresis loops, according to the IUPAC classification, indicating the presence of microporous activated carbon. The micropores were mostly distributed at <1.5 nm, as can be seen in Figure 4b. When the activation time was extended to 120 min at 850 °C, CS-850-120 showed some mesopores in the size range of 3–4 nm (Figure 4b) at a burn-off of 48.2%. The mesoporous volume, estimated from the adsorption isotherm, was less than 0.06 cm^3^/g (Table 3), confirming that these CS-based activated carbon materials were essentially microporous.

Figure 4c shows that the nitrogen adsorption/desorption isotherms of BC- and OC-based activated carbons exhibited a steep knee at relative pressures of <0.1 and a continuous upward trend at relative pressures of >0.1, indicating the formation of mesopores during the steam activation of these samples, even at a lower burn-off of around 35%. However, no hysteresis loops were apparent, revealing that the mesopores were distributed at <4 nm. Their pore distributions confirmed that these activated carbon samples had a pore structure that consisted mainly of micropores with just a few mesopores. For OC-850-60, the mesopore volume exceeded 30%, as shown in Table 3.

For FCs and MCs, steam activation yielded activated carbon samples with remarkably different pore structures compared to the other starting materials. Their nitrogen adsorption/desorption isotherms were characteristic of type IV, according to the IUPAC classification. Outstandingly, although FC-850-30 and MC-850-30 were obtained at a very low burn-off of 36.6% for FCs and 27.7% for MCs, they exhibited apparent hysteresis loops. This demonstrates that the steam activation of FCs and MCs can produce well-developed mesopore structures at a low burn-off, as confirmed by the pore-size distributions in Figure 4f. Table 3 shows that their mesopore volumes exceeded 30%, which is much higher than those of the other activated carbons.

The above results on the pore structures of steam-activated carbons demonstrate that the species of the lignocellulosic precursor has a significant effect on pore development in the process of steam activation at the initial stage of a low burn-off. The steam activation of CSs and BCs at low burn-off levels produced microporous activated carbon materials, whereas OCs, FCs, and MCs produced microporous/mesoporous activated carbon materials. Previously, it was believed that the physical activation of charcoal created micropores at a low burn-off level. Here, it is noted that BCs had a higher porosity than OCs or MCs (Table 2) and hence a high degree of the activation rate (Figure 3), but the former evolved into a predominantly microporous structure, while OCs and MCs produced well-developed mesoporous structures at the initial stage of steam activation. Therefore, the porosity of raw charcoals is not a decisive factor in determining the pore structure of steam-activated carbons. 

### 2.4. Pore Structures of Activated Carbons at High Burn-Off

**Figure 5:**
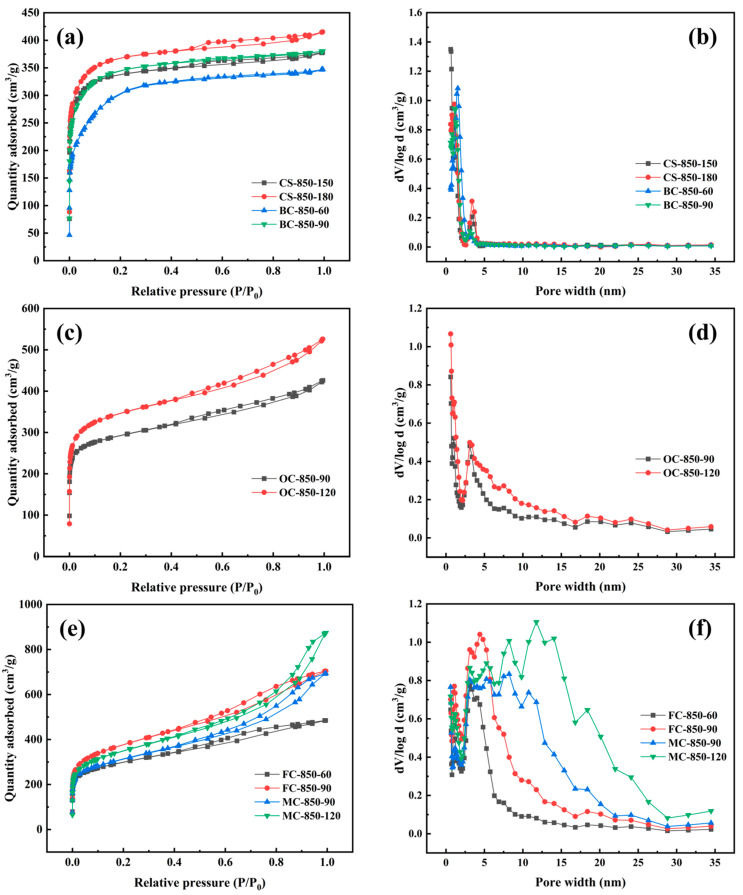
Nitrogen adsorption/desorption isotherms (**a**,**c**,**e**) and pore-size distributions (**b**,**d**,**f**) of activated carbons obtained at a high burn-off of >50%.

Figure 5 displays the nitrogen adsorption/desorption isotherms and pore-size distributions of the activated carbons obtained at a high burn-off of >50%. Their pore structure parameters are listed in Table 4. It can be seen that the pore structures of the activated carbon products changed greatly with the species of raw charcoal subjected to steam activation. Figure 5a shows that CS- and BC-based activated carbons presented type I isotherms when the burn-off was increased to 66.2% for CS-850-180 and 74.6% for BC-850-90. At a high burn-off level, micropore development was enhanced, while mesopores were poorly developed. With an increase in the burn-off, the micropore volumes of the activated carbons gradually increased, while their mesopore volumes were low, specifically 0.120 cm^3^/g for CS-850-180 and 0.117 cm^3^/g for BC-850-90 (Table 4). This suggests that the steam activation of CS- and BC-based charcoals at a high burn-off of >50% mainly produced micropores with a minority of mesopores, which was confirmed by their pore-size distributions (Figure 5b). The mesopores produced were stably distributed in a narrow range of 3–4 nm at different burn-offs, as shown in Figure 5b. For the OC-based charcoals, the activated carbon obtained at a high burn-off level maintained a similar adsorption isotherm to that obtained at a low burn-off level. This means that the steam activation of OCs produced micropores and mesopores in the high burn-off stage, as in the low burn-off stage. Table 4 shows that the development of both micropores and mesopores was gradually promoted by charcoal gasification at a burn-off of <80%; at around a 72% burn-off, the mesopore rate reached around 40%.

Figure 5e shows that the adsorption isotherms of FC- and MC-based activated carbons prepared at a high burn-off level were all of type IV, with distinct hysteresis loops, similar to those of materials prepared at a low burn-off. Clearly, their micropores and mesopores were progressively enhanced by an increased burn-off in the course of steam activation (Table 4). At a burn-off of 73.5% for FCs and 62.7% for MCs, the mesopore volumes of the obtained activated carbons were higher than their micropore volumes. The activated carbon prepared from MCs at a burn-off of 78.0% attained a high mesopore volume of 0.887 cm^3^/g, which is much higher than their micropore volume. More importantly, Figure 5f reveals that the pore-size distributions of the activated carbons were prominently broadened to wider ranges of 2–20 nm for FCs and 2–30 nm for MCs at a high burn-off. Therefore, the steam activation of FCs and MCs can evidently afford activated carbons with highly developed mesoporous structures.

Consequently, the steam activation of lignocellulose-based charcoals in a high burn-off regime can produce three kinds of activated carbons from these five raw charcoals, namely microporous, microporous/mesoporous, and highly developed mesoporous, depending on the species. At a high burn-off level, CSs and BCs produced predominantly microporous activated carbon with a microporosity of >80%; OCs produced microporous/mesoporous activated carbon; and FCs and MCs produced highly developed mesoporous activated carbon. The chemical compositions (ash and volatile matter), and the porosity of the raw charcoals (Table 1 and Table 2) does not provide a straightforward rationale for the decisive effect of the charcoal species on the pore-structure development at the initial and high burn-off stages of steam activation. We envisage that this effect is caused by the botanical structures of the charcoals inherited from the raw lignocelluloses. As illustrated in Figure 1, CSs exhibited a dense botanical texture, BCs had a high proportion of fiber bundle-derived dense char, OCs retained a hardwood-inherited botanical structure, and FCs and MCs maintained softwood-inherited botanical structures. Consequently, the OCs had plenty of tunnels originating from wood fibers and parenchyma cells, while FCs and MCs had considerable amounts of orderly arranged tracheid-derived tunnels. These tunnels were micrometer-sized with much thinner walls than those in dense CS and BC chars. We envisage that extensive tunnels with thinner walls in charcoals facilitate the formation of mesopores by simultaneously gasifying carbon atoms from two sides of the thin walls. The thinner the walls of these tunnels, the more easily mesopores can be formed at a lower burn-off in the process of physical activation. As a result, FCs and MCs produced initially developed mesopores at a lower burn-off level and highly developed mesopores at a high burn-off. It is noteworthy that the microporous activated carbon prepared from CSs had a higher surface area than the microporous/mesoporous or mesoporous activated carbons prepared from OCs, FCs, and MCs, even with approximately the same burn-off value. This is because most of the added pore surface in the activated carbon originates from the generation of micropores during the physical activation process.

### 2.5. Vitamin B12 Adsorption on Steam-Activated Carbons

**Figure 6:**
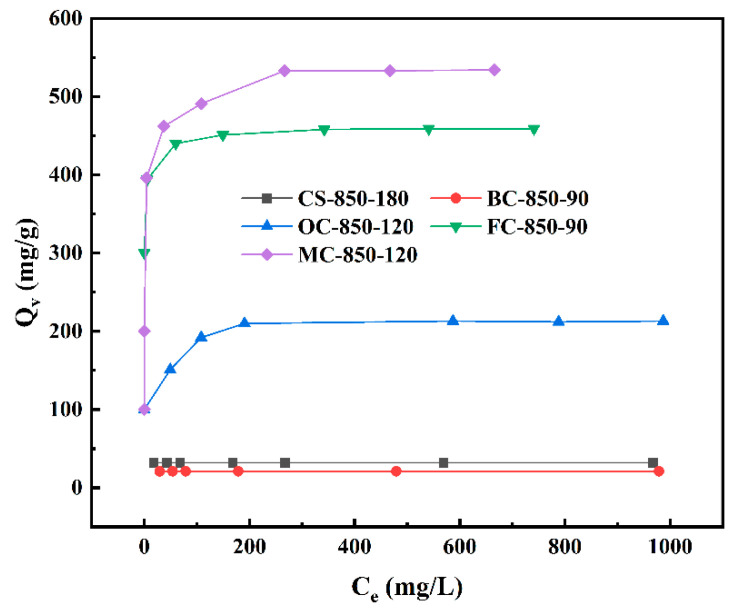
Adsorption isotherms of coconut shell, bamboo, sawtooth oak, Masson pine, and Chinese fir activated carbons toward VB12.

In order to further explore the differences in the pore structure of activated carbons prepared from different lignocellulosic raw materials and the effects of pore structure upon the adsorption property, we applied the activated carbon materials to VB12 adsorption. Figure 6 presents the adsorption isotherms of VB12 for CS, BC, MC, FC, and OC activated carbons obtained at a burn-off of above 66%. The results show that the sample MC-850-120 displayed an adsorption capacity for VB12 with a value of as high as ~530 mg/g, which is higher than that of some reported mesoporous materials [40,41,42,43]. Similarly, FC-850-90 showed a high VB12 adsorption capacity of ~460 mg/g. These high adsorption capacities toward VB12 further confirm the highly developed mesopores of 5–20 nm of these steam-activated carbons prepared from FCs and MCs at a high burn-off (Figure 5f). The molecular size of VB12 is 2.09 nm, and its molecular weight is 1355 Da [43]; thus, the pores that efficiently adsorb VB12 molecules must be of a size greater than 2.09 nm [44]. The samples CS-850-180 and BC-850-90 had only a few mesopores with sizes mainly concentrated in the range of 3–4 nm and hence exhibited a much lower adsorption capacity of ~30 mg/g and ~20 mg/g, respectively. OC-850-120 also showed a much lower adsorption capacity toward VB12, ~210 mg/g, because of its lower mesopore volume, with mesopores smaller than those in MC-850-120 and FC-850-90.

## 3. Experimental Section

### 3.1. Preparation of Activated Carbon Materials

In this study, Masson pine (Pinus massoniana) charcoals (MCs), Chinese fir (Cunninghamia lanceolata) charcoals (FCs), sawtooth oak (Quercus acutissima) charcoals (OCs), coconut (Cocos nucifera) shell charcoals (CSs), and Moso bamboo (Phyllostachys edulis) charcoals (BCs) were selected for steam activation. They were prepared by carbonizing at temperatures in the range of 600–800 °C for 150 min in a high temperature carbonization furnace. Prior to activation, the charcoals were crushed and sieved to obtain particles with a size of 1.40–3.35 mm. Steam activation was carried out in a horizontal high-temperature rotary furnace with programmable heating steps. Typically, weighed charcoal was placed in the furnace and heated to 850 °C at a rate of 10 °C/min under a nitrogen flow of 200 mL/min. The nitrogen was then turned off, and a steam flow of 300 mL/min was then introduced into the reactor to activate the charcoal for a given duration of 30–180 min. Finally, the activated samples were cooled to an ambient temperature under a nitrogen atmosphere. The activated carbons of CSs, OCs, BCs, FCs, and MCs are designated as CS-X-Y, OC-X-Y, BC-X-Y, FC-X-Y, and MC-X-Y, respectively, where X denotes the activation temperature (°C), and Y denotes the activation time (min). The activation burn-off was determined according to Equation (1):

(1) $$Burn-off\;\left(\%\right)=\frac{M_{0}-M}{M_{0}}\times 100\%$$

where *M*_0_ and *M* are the masses (g) of carbons before and after activation, respectively.

### 3.2. Characterization

Proximate analyses of moisture, ash, and volatile matter were conducted according to the Chinese national standards GB/T 12496.4-1999 [45], GB/T 12496.3-1999 [46], and GB/T 17664-1999 [47], respectively. Ultimate analyses were obtained by using an elemental analyzer (Vario EL Cube, Elementar, Hanau, Germany), and oxygen contents were determined according to the oxygen model.

Nitrogen adsorption–desorption isotherms of the activated carbon samples were measured with an Autosorb-iQ2 nitrogen adsorption instrument (Quantachrome, Boynton Beach, FL, USA) at −196 °C. Before measurement, each sample was degassed at 175 °C for 10 h. The surface area (S_BET_) was calculated by the Brunauer–Emmett–Teller (BET) method. The total pore volume (V_tot_) was estimated from the volume of liquid nitrogen which is equivalent to the volume of adsorbed nitrogen at a relative pressure of 0.99. The micropore volume (V_mic_) was calculated according to the Dubinin–Radushkevic (DR) equation. The mesopore volume (V_mes_) was obtained by subtracting V_mic_ from V_tot_. The pore-size distribution was evaluated by the quenched-solid density functional theory (QSDFT). The morphologies of the charcoal samples were examined by environmental scanning electron microscopy (ESEM) using a Quanta200 instrument (FEI, OR, USA) that was operated at an accelerating voltage of 15 KV.

Mercury injection porosimetry (MIP) was performed using an AutoPore V 9600 instrument (Micromeritics Instrument Corporation, GA, USA) to elucidate the textural properties of the charcoal samples, including the pore volume, porosity, and pore-size distribution. The pore-size distribution was determined according to the Washburn equation, as shown in Equation (2) [31,48]:

(2) $$r=-\frac{2\gamma \cdot cos\theta }{P}$$

where *r* is the pore radius (nm), *p* is the pressure (MPa), *γ* = 0.48 N/m (the surface tension of mercury), and *θ* = 140° (the contact angle between mercury and the measured charcoal samples).

### 3.3. Vitamin B12 Adsorption

The vitamin B12 (VB12) was analyzed a using UV-2600 spectrophotometer (Shimadzu, Japan), with a detected characteristic peak at 361 nm [49]. Adsorption tests were performed by adding 0.100 g of each activated carbon sample to aliquots (100 mL) of VB12 solutions at concentrations ranging from 100 mg/L to 1200 mg/L in a series of 250 mL stoppered conical flasks. The mixtures were shaken at 160 rpm in a water bath maintained at 25 °C with protection from light for 24 h. The adsorbed amounts were calculated according to Equation (3):

(3) $$Q_{V}=\frac{\left(C_{0}-C_{e}\right)V}{W}$$

where *Q_V_* is the adsorption capacity (mg/g), *C*_0_ and *C_e_* are the initial and equilibrium concentrations (mg/L) in the solution, *V* is the volume (mL) of the solution, and *W* is the mass (g) of the activated carbon sample.

## 4. Conclusions

Five representative lignocellulosic starting materials, namely coconut shells, bamboo, sawtooth oak, Chinese fir, and Masson pine, were selected for the production of activated carbon by steam activation in an effort to investigate the effects of the species of raw materials on physical activation. The raw charcoals were characterized by proximate and ultimate analyses, ESEM, and mercury injection porosimetry. A series of activated carbons was prepared from these five charcoals by steam activation for different durations. The pore structures of these activated carbon materials were analyzed by recording nitrogen adsorption isotherms. It was found that the porosity of a raw charcoal determines the rate of its reaction with steam in the activation process. An inspection of the pore structure showed that the original textures of the lignocellulosic raw materials have a decisive effect on the pore-structure development of the obtained steam-activated carbons. The steam activation of coconut shells and bamboo charcoals mainly produced micropores at the whole burn-off range of 0–80% and thus afforded predominantly microporous activated carbon materials with just a few mesopores at a high burn-off of >50%. The steam activation of oak charcoals produced mainly micropores at a low burn-off of <50% but both micropores and mesopores at a high burn-off of >50%, thus affording microporous/mesoporous activated carbon materials at a high burn-off. Notably, the steam activation of Chinese fir and Masson pine charcoals produced mainly mesopores at the whole burn-off range of 0–80%, and the size of these mesopores was remarkably increased to 10–25 nm by extending the activation time. At a high burn-off, Chinese fir and Masson pine charcoals provided highly developed mesoporous activated carbons, and Masson pine activated carbons have an adsorption capacity as high as 530 mg/g, toward the large-sized molecule VB12.

## Figures and Tables

**Table 1:** Proximate and ultimate analyses of charcoals.

Samples	Proximate Analysis (%)				Ultimate Analysis (%)					C/N
	Moisture	Ash	Volatile Matters	Fixed Carbon	C	H	N	O	S	
CS	5.40	1.63	21.75	76.62	90.94	3.39	3.52	1.97	0.17	25.84
MC	2.69	1.47	7.11	91.42	90.50	3.16	3.64	2.51	0.17	24.86
FC	1.22	0.72	6.66	92.62	90.72	3.52	3.62	1.99	0.12	25.06
OC	5.76	3.86	27.35	68.79	88.49	4.33	3.87	3.13	0.15	22.87
BC	5.40	4.26	6.78	88.96	88.00	2.44	3.83	5.57	0.14	22.98

**Table 2:** Pore structure parameters from mercury intrusion data of charcoals.

Samples	Total Intrusion Volume (mL/g)	Average Pore Diameter (nm)	Bulk Density (g/mL)	Porosity (%)
CS	0.3326	59.58	0.8751	29.10
OC	1.0912	115.81	0.5201	56.76
BC	2.5339	189.23	0.2771	70.21
FC	3.4573	2553.83	0.2191	75.77
MC	1.6990	1719.94	0.3816	64.83

**Table 3:** Pore parameters of activated carbons obtained at a low burn-off.

Samples	Burn-Off (%)	S_BET_ (m^2^/g)	V_tot_ (cm^3^/g)	V_mic_ (cm^3^/g)	V_mes_ (cm^3^/g)	Mesopore Rate (%)
CS-850-30	19.8	701	0.304	0.268	0.036	11.84
CS-850-60	25.0	816	0.346	0.312	0.034	9.83
CS-850-90	38.8	861	0.372	0.331	0.041	11.02
CS-850-120	48.2	1162	0.505	0.446	0.059	11.68
OC-850-30	35.3	639	0.304	0.242	0.062	20.39
OC-850-60	49.7	1011	0.562	0.388	0.174	30.96
BC-850-30	36.7	873	0.391	0.334	0.057	14.58
FC-850-30	36.6	820	0.448	0.313	0.135	30.13
MC-850-30	27.7	691	0.403	0.264	0.139	34.49
MC-850-60	48.5	855	0.572	0.326	0.246	43.01

**Table 4:** Pore parameters of activated carbons obtained at a high burn-off.

Samples	Burn-Off (%)	S_BET_ (m^2^/g)	V_tot_ (cm^3^/g)	V_mic_ (cm^3^/g)	V_mes_ (cm^3^/g)	Mesopore Rate (%)
CS-850-150	58.4	1310	0.585	0.491	0.094	16.07
CS-850-180	66.2	1415	0.642	0.522	0.120	18.69
OC-850-90	54.5	1110	0.659	0.425	0.234	35.51
OC-850-120	71.7	1301	0.814	0.493	0.321	39.43
BC-850-60	59.0	1122	0.537	0.361	0.176	32.77
BC-850-90	74.6	1312	0.588	0.471	0.117	19.90
FC-850-60	58.0	1090	0.750	0.411	0.339	45.20
FC-850-90	73.5	1360	1.089	0.501	0.588	53.99
MC-850-90	62.7	1122	1.073	0.481	0.592	55.17
MC-850-120	78.0	1260	1.352	0.465	0.887	65.61

## Data Availability

The data presented in this study are available on request from the corresponding author.
